# Associations between ICT-related digital teaching demands and job burnout among university physical education teachers in China: the intervening roles of technostress and occupational stress

**DOI:** 10.3389/fpsyg.2026.1842215

**Published:** 2026-07-06

**Authors:** Fei Yang, Wangjie Li, Chen Li

**Affiliations:** 1 School of Physical Education, Nanyang Normal University, Nanyang, China; 2 College of Sports and Great Health, Sichuan Technology and Business University, Meishan, China

**Keywords:** ICT-related digital teaching demands, job burnout, occupational stress, technology-related stress, university physical education teachers

## Abstract

**Purpose:**

This study examined the cross-sectional association between ICT-related digital teaching demands and job burnout among university physical education (PE) teachers, and assessed whether technology-related stress and occupational stress served as stress-related pathways linking these demands to burnout.

**Methods:**

A questionnaire survey was conducted among university PE teachers from Henan Province, China, yielding 1,869 valid responses. Digital teaching demands were operationalized as ICT-related work demands. Structural equation modeling was used to estimate the proposed associations, and indirect pathways were tested using bias-corrected percentile bootstrapping with 5,000 resamples.

**Results:**

ICT-related digital teaching demands were positively associated with job burnout. The direct association remained significant after accounting for technology-related stress and occupational stress, but the indirect component accounted for a larger proportion of the total association. The total indirect association was significant [β = 0.292, 95% CI (0.254, 0.330)], indicating that stress-related pathways explained 68.54% of the total association. Significant indirect pathways were observed through technology-related stress, through occupational stress, and through the sequential pathway from technology-related stress to occupational stress.

**Conclusion:**

In this sample of university PE teachers from Henan Province, ICT-related digital teaching demands were positively associated with job burnout, and this association was largely reflected through technology-specific strain and broader occupational pressure. These cross-sectional findings suggest that digitally intensified teaching environments may be better understood by considering both technostress and general occupational stress, while causal interpretations should be tested in future longitudinal research.

## 1 Introduction

Digital transformation has become a defining feature of contemporary higher education. Learning management systems (Mella-Norambuena et al., 2025), classroom capture (Banerjee, 2021), online assessment platforms (dos Reis and America, 2024), data dashboards (Vanermen et al., 2025), mobile applications (Briz-Ponce et al., 2017), and AI-enabled tools (Ilieva et al., 2025) are increasingly embedded in curriculum delivery, teaching evaluation, and quality assurance. In many universities, digital competencies are no longer treated as optional add-ons; they are institutionalized as formal expectations through course construction projects (Graham et al., 2013), blended teaching requirements (Ali and Georgiou, 2025), and evidence-based appraisal processes (Porter et al., 2014; Santos et al., 2025). Under such conditions, teaching work is progressively reorganized around digital infrastructures (Schroeder et al., 2025), raising a practical question for academic management and educational research: when digital teaching requirements intensify, what happens to teachers’ occupational wellbeing?

University physical education (PE) teachers represent a particularly relevant group for examining that question. PE teaching relies heavily on embodied demonstration, real-time observation of movement, risk management, and on-site group organization. Digitalization introduces new layers of work into those already demanding instructional contexts (Liao et al., 2025). Routine tasks may expand to include producing and editing instructional videos, managing online course spaces, responding to platform-based student queries across extended hours, integrating wearable or app-based activity data into teaching and grading, uploading evidence for teaching inspections, and adapting to frequent updates of software and institutional systems (Jastrow et al., 2022). The coexistence of physical-space instruction and digital-space requirements can reshape workload patterns and time boundaries, potentially amplifying strain.

Job burnout remains one of the most consequential outcomes of sustained occupational strain in education. Burnout commonly manifests as emotional exhaustion, cynical or detached attitudes toward work, and a diminished sense of accomplishment (Kinman, 2024; Mihailescu et al., 2018; Maslach et al., 2001). Among PE teachers, burnout is not only an individual health concern; it can undermine instructional safety (Cordova et al., 2023; Descoeudres et al., 2022; Bartholomew et al., 2014; Lopez, 2026; Neto et al., 2024), reduce feedback quality (Abós et al., 2019; Haerens et al., 2019), weaken student motivation (García-González et al., 2019), and erode sustained participation in physical activity programs (Cid et al., 2019; Franco et al., 2025; Ran, 2024). In PE classes, for example, emotionally exhausted teachers may have less attentional capacity to monitor students’ movement techniques, detect safety risks, or provide timely corrective feedback during high-intensity activities. Cynical or detached attitudes may also reduce encouragement, individualized guidance, and the design of adaptive learning tasks that sustain students’ motivation and participation. For universities seeking to promote student health and to improve teaching quality, understanding how digital teaching demands are associated with burnout is therefore important for informing workload allocation, teacher training, technical support, platform governance, and evaluation policies.

Digital teaching demands do not automatically produce negative outcomes. Digital tools can enhance instructional effectiveness, diversify pedagogical resources, and improve formative assessment (Odoom et al., 2025). The critical issue concerns the balance between demands and support, along with the stress processes activated when teachers perceive insufficient time, skills, autonomy, or technical reliability to meet those demands (Kärner and Höning, 2021; Nascimento et al., 2025; Tu et al., 2025). When digital requirements exceed available resources, digital tools may shift from being instructional supports to becoming sources of strain. Two stress-related constructs appear especially useful for clarifying this process: technostress (Brod, 1984) and occupational stress (Kahn et al., 1964).

Technostress refers to stress experienced in relation to the use of digital technologies (Brod, 1984), often arising from technology overload, invasion into non-work time, complexity of systems, insecurity about competence or job standing, and uncertainty caused by frequent changes (Ragu-Nathan et al., 2008). In university settings, technostress may emerge when multiple platforms coexist without integration, when institutional requirements change quickly, or when technical support lags behind policy ambition (Gosse et al., 2024). For PE teachers, technostress can be intensified by the need to translate movement-based instruction into digital formats and by the need to operate devices and software under tight class schedules or outdoor teaching conditions (Lee et al., 2025; Zha et al., 2025).

Occupational stress reflects broader perceptions of work pressure, including role overload, time pressure, role conflict, administrative burden, and accountability demands (Kyriacou and Sutcliffe, 1978; Rushing, 1965). Digital teaching requirements may increase occupational stress by expanding the scope of responsibilities and compressing time for lesson preparation, training, research, and service. Even when technology functions well, work pressure may rise if digital tasks are layered on top of existing obligations rather than replacing them. When occupational stress remains high and recovery opportunities remain limited, burnout risk escalates (Sonnentag and Fritz, 2015).

Despite growing attention to digitalization in education, several gaps motivate a focused inquiry into university PE teachers. Research on teacher wellbeing under digital transformation often centers on general faculty populations or on school teachers, while the instructional ecology of PE differs in pedagogical form, safety demands, and assessment practices. In addition, studies sometimes treat technology-related stress as a single undifferentiated pressure or focus on direct associations between digital practices and wellbeing outcomes. Less clarity exists regarding pathways that simultaneously account for technology-specific strain and the broader experience of work pressure as connected processes. An integrated perspective that positions technostress and occupational stress as key stress-related pathways can improve explanatory precision and support more context-sensitive understanding of digitalized PE teaching (Gimpel et al., 2025).

The present study therefore examines whether technology-related stress and occupational stress mediate the association between digital teaching demands and job burnout among university PE teachers in Henan Province, China. Digital teaching demands are conceptualized as the perceived level of digital-related requirements and expectations embedded in teaching work. Technostress is treated as a proximal stress response triggered by technology use and technology governance conditions, whereas occupational stress is treated as a broader appraisal of work pressure within the job environment. The model evaluates whether digital teaching demands are associated with burnout through technostress, through occupational stress, and through a connected pathway in which technology-related strain is associated with broader occupational pressure.

## 2 Literature review and hypotheses

### 2.1 Digital teaching demands as a digital job demand

Digital teaching demands refer to the extent to which teaching work is accompanied by expectations for technology-enabled course delivery, platform-based interaction, digital resource development, data reporting, and compliance with digitally mediated evaluation requirements. In the present study, this broad construct is empirically operationalized as ICT-related digital work demands, emphasizing communication overload, accessibility expectations, technology-based interruptions, and work disruption caused by malfunctioning digital equipment. This operationalization captures an important ICT-demand component of digitalized teaching work, while recognizing that digital teaching in PE may also involve discipline-specific practices such as video production, wearable-data management, and platform-based assessment.

Within the job demands–resources (JD–R) perspective, demands denote aspects of work that require sustained physical, cognitive, or emotional effort and therefore tend to be associated with physiological or psychological costs when adequate resources are unavailable (Bakker and Demerouti, 2007; Demerouti et al., 2001). Digital teaching demands can be understood as a contemporary form of job demand because they extend teachers’ responsibilities beyond face-to-face instruction into digitally mediated communication, documentation, evaluation, and data management. This does not imply that digital technologies are inherently harmful. Rather, from the JD–R perspective, digital requirements become more likely to generate strain when they intensify work without corresponding increases in time, autonomy, technical support, or digital competence.

For university PE teachers, this demand structure may be particularly salient. PE teaching is not only knowledge-based but also embodied, interactive, and safety-sensitive. Teachers must demonstrate movements, observe students’ physical performance, manage group activities, prevent injury, and provide immediate feedback in dynamic teaching spaces. When digital requirements are layered onto these responsibilities, teachers may need to prepare online materials, upload teaching evidence, respond to platform-based messages, process activity or fitness data, and adapt to changing institutional systems. Such demands may increase perceived workload, fragment attention, and reduce opportunities for psychological recovery. In this sense, ICT-related digital teaching demands may represent a digital extension of the health-impairment process described in the JD–R model.

Burnout is a major outcome of prolonged exposure to excessive job demands. It is commonly characterized by emotional exhaustion, depersonalization or cynical attitudes toward work, and reduced personal accomplishment (Maslach et al., 2001). When digital teaching demands consume time and energy while narrowing teachers’ perceived control over work pace and boundaries, they may be associated with higher burnout risk. Although job and personal resources such as digital competence, organizational support, autonomy, and technical assistance may buffer this process, the present study focuses specifically on the demand–stress pathway as a first step toward understanding the health-impairment route in digitally intensified PE teaching contexts.

*H1:* Digital teaching demands are positively associated with job burnout among university physical education teachers.

### 2.2 Technology-related stress as a mediating mechanism

Technology-related stress, or technostress, describes strain arising from the use and governance of digital technologies, including experiences of technology overload, complexity, invasion into non-work time, insecurity, and uncertainty (Brod, 1984; Tarafdar et al., 2007). Conceptually, technostress helps distinguish between the presence of digital demands and teachers’ stress appraisal of those demands. Digital systems may be introduced as tools for efficiency and instructional innovation, but they can become stressors when teachers experience them as excessive, unreliable, intrusive, difficult to master, or poorly aligned with their teaching tasks.

In higher education, intensified digital requirements may increase the frequency, breadth, and urgency of technology use. Teachers may be expected to manage learning platforms, respond to online messages, complete digital documentation, adapt to system updates, and demonstrate evidence of teaching quality through platform-based indicators. These requirements can raise the probability of fatigue and frustration when platforms are fragmented, updates are frequent, or technical support is limited. For PE teachers, additional sources of technostress may include the challenge of translating embodied demonstrations into digital formats, collecting or interpreting activity data, using devices in outdoor or venue-based classes, and managing multi-channel communication with students beyond scheduled class time.

A growing body of teacher-focused technostress research supports the relevance of this pathway. Wang and Li (2019) examined university teachers and proposed technostress as a multidimensional person–environment misfit, suggesting that stress emerges when teachers perceive a mismatch between technological demands and their abilities, resources, or instructional context. Solís et al. (2023) further showed that perceived organizational support is closely related to teachers’ technostress, indicating that technology-related strain is shaped not only by individual competence but also by institutional conditions. Özgür (2020) also showed that teachers’ technostress is related to TPACK, school support, and demographic variables. Levante et al. (2025) found that technostress was involved in the pathway linking coping, online-teaching self-efficacy, and emotional exhaustion among teaching staff during the COVID-19 period. A recent systematic review by Yang D. et al. (2025) also emphasized that teacher technostress research has expanded rapidly and that future studies should pay closer attention to its sources, outcomes, and mitigating conditions.

These studies suggest that technostress is not merely a technical problem; it is a work-design and resource-alignment issue. In the present context, digital teaching demands may increase teachers’ exposure to communication overload, constant reachability, technical interruptions, and platform complexity. These experiences may then be appraised as technology-related stress, which is likely to be associated with burnout symptoms such as emotional exhaustion and reduced efficacy. Prior work on technostress also indicates meaningful links with exhaustion, reduced satisfaction, and impaired wellbeing, suggesting a plausible pathway connecting digital teaching demands with burnout outcomes (Pflügner et al., 2024; Ragu-Nathan et al., 2008; Tarafdar et al., 2007). Therefore, technology-related stress is expected to mediate the association between digital teaching demands and job burnout.

*H2:* Technology-related stress mediates the association between digital teaching demands and job burnout among university physical education teachers.

### 2.3 Occupational stress as a mediating mechanism

Occupational stress reflects the perceived pressure arising from broader work conditions such as role overload, time constraints, role conflict, administrative burden, student-related demands, professional development pressure, and accountability requirements. From stress appraisal perspectives, perceived stress depends on how work demands are evaluated relative to available resources, autonomy, and support. Whereas technostress captures strain specifically related to digital technologies, occupational stress captures a more general appraisal of work pressure within the job environment.

Digital teaching demands may contribute to occupational stress because they often expand the scope of teaching work. A digital task rarely exists in isolation. Preparing online resources may require additional planning time; managing platform-based interaction may extend teachers’ availability beyond formal working hours; uploading teaching evidence may increase administrative burden; and digital assessment or data reporting may intensify accountability. Even when technology functions well, work pressure may rise if digital tasks are added to existing teaching, research, service, and student-management responsibilities rather than replacing them.

This issue may be especially relevant for university PE teachers. PE teaching already involves physically intensive instruction, class organization, safety management, venue coordination, and individualized feedback. When digital requirements are added, teachers may experience a broader sense of role expansion: they are expected not only to teach physical movement but also to document, digitize, analyze, and report that teaching through institutional systems. Such expansion can increase perceived workload and time pressure, which are central components of occupational stress. Over time, persistent occupational stress may be associated with burnout symptoms, particularly emotional exhaustion and cynicism (Maslach et al., 2001).

Accordingly, occupational stress provides a second pathway through which digital teaching demands may be associated with job burnout. In this pathway, digital teaching demands are not assumed to produce burnout directly and uniformly. Rather, they may become burnout-relevant when teachers appraise them as contributing to broader work pressure, insufficient recovery, role overload, and reduced control over work boundaries.

*H3:* Occupational stress mediates the association between digital teaching demands and job burnout among university physical education teachers.

### 2.4 Sequential mediation via technology-related stress and occupational stress

Technology-related stress and occupational stress are conceptually distinct but potentially connected. Technostress can remain technology-specific, such as frustration with complex platforms, equipment malfunction, or difficulty managing online communication. However, it can also spill over into broader occupational stress when technology-related problems delay other tasks, extend working time, disrupt teaching preparation, or reduce teachers’ perceived control over work pace. In this sense, technology-related stress may serve as a proximal response to digital teaching demands, whereas occupational stress may reflect a broader downstream appraisal of accumulated work pressure.

Conservation of resources theory suggests that resource loss tends to accumulate and that strain in one domain can reduce individuals’ capacity to cope with demands in another domain (Hobfoll, 1989; Hobfoll et al., 2018; Tarafdar et al., 2007). Applied to digitally intensified teaching, teachers who spend time and cognitive energy troubleshooting platforms, responding to online messages, adapting to software changes, or converting PE content into digital formats may have fewer resources available for lesson preparation, student interaction, research tasks, and recovery. Technology-related strain can therefore contribute to a broader sense of occupational pressure through time depletion, cognitive fatigue, boundary disruption, and perceived loss of control.

For university PE teachers, this sequential process may be particularly meaningful because digital work and embodied teaching work are often not substitutes. Instead, they coexist. A teacher may conduct in-person physical instruction during the day, then process digital records, respond to platform messages, or prepare online materials after class. In such circumstances, technostress may be associated with increased occupational stress because technology-related difficulties make the entire work system feel more demanding. When occupational stress intensifies and recovery opportunities remain limited, burnout risk may rise.

This reasoning supports a sequential mediating pathway. Digital teaching demands may first be associated with technology-related stress; technology-related stress may then be associated with broader occupational stress; and occupational stress may subsequently be associated with job burnout. This pathway helps explain how a specific form of digital strain may become embedded in a more general process of occupational pressure and burnout.

*H4*: Technology-related stress and occupational stress sequentially mediate the association between digital teaching demands and job burnout among university physical education teachers.

### 2.5 Theoretical model

**FIGURE 1:**
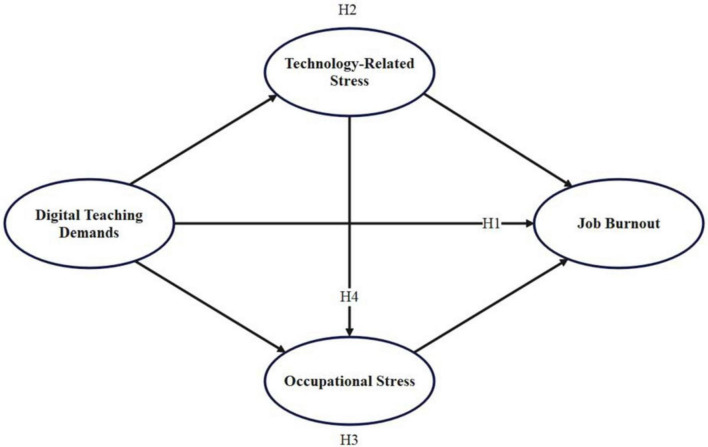
Proposed theoretical model linking digital teaching demands to job burnout through technology-related stress and occupational stress. H1 represents the direct association between digital teaching demands and job burnout. H2 and H3 represent the two parallel indirect pathways through technology-related stress and occupational stress, respectively. H4 represents the sequential indirect pathway through technology-related stress and occupational stress. All hypothesized associations are positive.

Figure 1 presents the proposed theoretical model. Digital teaching demands are hypothesized to be positively associated with job burnout directly (H1). In addition, technology-related stress is proposed as a mediating pathway linking digital teaching demands to job burnout (H2), while occupational stress is proposed as a second mediating pathway (H3). The model further specifies a sequential mediation pathway in which digital teaching demands are associated with technology-related stress, technology-related stress is associated with occupational stress, and occupational stress is associated with job burnout (H4). Thus, the model captures both parallel and sequential stress-related pathways. All hypothesized associations are expected to be positive.

## 3 Materials and methods

### 3.1 Participants and data

The target population for this study consisted of physical education teachers from 10 universities in Henan Province, China. Data were collected through an online questionnaire administered from December 1 to December 15, 2025. The survey link was distributed through professional and institutional channels, with participation being voluntary and anonymous. Before completing the questionnaire, participants were informed of the purpose of the study, the voluntary nature of participation, the anonymous treatment of responses, and their right to withdraw at any time. Online informed consent was obtained from all participants.

The research protocol was reviewed and approved by the Ethics Committee of Nanyang Normal University, China (Approval No. 2025NYNU050). A total of 2,131 questionnaires were returned. To ensure response quality, data screening procedures were applied. Questionnaires were excluded if they contained substantial missing data, exhibited abnormally short completion times suggesting inattentive responses, displayed clear straight-line answering patterns across most items, or included logically inconsistent information. After removing 262 invalid responses, 1,869 valid questionnaires remained, yielding an effective response rate of 87.71%. The original anonymized data supporting the findings of this study are provided in the Supplementary material.

Sample size adequacy was evaluated based on established empirical guidelines for survey research and structural equation modeling, which recommend a minimum sample size of 15–20 times the total number of measurement items (Kline, 2023). The study included 64 items in total, comprising 6 items for Digital Teaching Demands, 19 items for Technology-Related Stress, 22 items for Occupational Stress, and 17 items for Job Burnout. According to the 15–20 times rule, the recommended sample size ranged from 960 to 1,280. With 1,869 valid responses, the final sample size exceeded the upper bound of this range, thus meeting commonly accepted methodological requirements for subsequent analyses.

### 3.2 Measurement tools

All constructs were measured using five-point Likert-type scales, with response options ranging from 1 (strongly disagree) to 5 (strongly agree). Higher scores indicated higher levels of the corresponding construct.

Digital Teaching Demands were assessed using the six-item ICT Demands at Work scale (SLOSH version) (Stadin et al., 2019). In this study, digital teaching demands were operationalized as ICT-related work demands embedded in teaching activities. The scale conceptualizes ICT-related requirements as a specific form of job demand, capturing communication overload, accessibility expectations, task interruption, and work disruption caused by equipment malfunction. It is commonly treated as a unidimensional construct. A representative item is: “I receive too many calls and emails at work.” In the present study, the Cronbach’s α coefficient for this scale was 0.878. Although this scale captures the ICT-demand component of digitalized teaching work, it does not cover all PE-specific digital tasks, such as instructional-video production, wearable-data management, or real-time activity-data processing. This operational boundary was considered when interpreting the findings.

Technology-Related Stress was measured using the Teacher Information Technology Stress Scale developed by Du (2013). The instrument consists of 19 items across three dimensions: increased workload, technology invasion into personal life, and technology complexity. A representative item is: “New technologies are too complex for me to understand and use.” In the present sample, the Cronbach’s α coefficients for the three subdimensions ranged from 0.861 to 0.930.

Occupational Stress was assessed using the Teacher Job Stress Questionnaire developed by Li et al. (2011). The instrument contains 22 items across five dimensions: workload stress, student academic pressure, social and school evaluation pressure, professional development pressure, and student problem behavior pressure. A representative item is: “Differences among students and their uneven learning levels make me feel stressed.” In this study, the Cronbach’s α coefficients for the subdimensions ranged from 0.807 to 0.900.

Job Burnout was measured using the Teacher Burnout Questionnaire developed by Zheng (2013). The instrument includes 17 items across three dimensions: emotional exhaustion, depersonalization, and reduced personal accomplishment. A representative item is: “I feel emotionally drained from my work.” In the present study, the Cronbach’s α coefficients for the three subdimensions ranged from 0.868 to 0.889.

Because the Occupational Stress and Job Burnout instruments were originally developed in teacher samples that included school-teaching contexts, the wording of relevant items was reviewed and adapted for the university PE teaching context before formal administration. Context-specific expressions related to school settings, teaching evaluation, student learning, and professional development were adjusted where necessary to ensure applicability to university PE teachers. These adaptations retained the substantive meaning of the original items and did not alter the response format or construct dimensions.

### 3.3 Data analysis

All statistical analyses were conducted using IBM SPSS Statistics 26.0 and AMOS 26.0. The analyses followed standard multivariate data-analysis procedures (Ziegel, 1988). Prior to hypothesis testing, preliminary data screening was performed to examine missing values, outliers, and distributional properties. Invalid cases had already been removed during the data cleaning stage. Skewness and kurtosis values for all variables were within acceptable ranges, supporting the use of maximum likelihood estimation in subsequent analyses (Kline, 2023).

Descriptive statistics were calculated for Digital Teaching Demands, Technology-Related Stress, Occupational Stress, and Job Burnout. Internal consistency reliability was assessed using Cronbach’s α. Composite reliability (CR) and average variance extracted (AVE) were computed to evaluate construct reliability and convergent validity. Convergent validity was considered adequate when CR exceeded 0.70, AVE exceeded 0.50, and standardized factor loadings were above 0.60 (Ashley, 2025; Fornell and Larcker, 1981).

Confirmatory factor analysis (CFA) was conducted to test the measurement model including four latent variables: Digital Teaching Demands, Technology-Related Stress, Occupational Stress, and Job Burnout. The indicator specification was determined a priori according to the measurement structure of each scale. Digital Teaching Demands was modeled using its six original items as observed indicators because the scale is unidimensional. Technology-Related Stress, Occupational Stress, and Job Burnout were modeled using their theoretically defined subdimension mean scores as observed indicators. Specifically, Technology-Related Stress was represented by three dimension scores, Occupational Stress by five dimension scores, and Job Burnout by three dimension scores. Thus, the CFA and SEM models were estimated using 17 observed indicators in total. This specification was based on the theoretical dimensionality of the instruments rather than on data-driven item deletion or *post-hoc* model modification.

Model fit was evaluated using multiple indices, including the chi-square to degrees of freedom ratio (χ^2^/df) (Marsh and Hocevar, 1985), Comparative Fit Index (CFI), Tucker–Lewis Index (TLI), Standardized Root Mean Square Residual (SRMR), and Root Mean Square Error of Approximation (RMSEA) with its 90% confidence interval (Browne and Cudeck, 1992; Hu and Bentler, 1999). Alternative measurement models, including one-factor, two-factor, three-factor, and four-factor models, were compared to examine discriminant validity (Anderson and Gerbing, 1988). No *post-hoc* correlated residuals, cross-loadings, or modification-index-based adjustments were added to force model fit.

Given that all variables were measured through self-report questionnaires, common method bias was assessed using both exploratory and confirmatory approaches. Harman’s single-factor test was first performed through unrotated exploratory factor analysis to examine whether a single factor accounted for the majority of covariance (Podsakoff et al., 2003; Podsakoff and Organ, 1986). Subsequently, a CFA-based comparison between the hypothesized four-factor model and a single-factor model was conducted. An unmeasured latent method construct model was also estimated by specifying a latent common method factor loading on all observed indicators. Common method bias was considered unlikely to dominate the observed associations when the first unrotated factor explained < 40% of the total variance and when the method factor model did not show a substantially improved fit relative to the hypothesized model (Williams et al., 2010).

Pearson correlation analyses were conducted to examine bivariate associations among the four constructs (Cohen, 1988). To test the hypothesized mediation framework, structural equation modeling was performed in AMOS 26.0, specifying both direct and indirect paths from Digital Teaching Demands to Job Burnout through Technology-Related Stress and Occupational Stress. The structural model used the same indicator specification as the CFA model and was evaluated using the same fit indices described above. Indirect associations were examined using the bias-corrected bootstrap method with 5,000 resamples (Shrout and Bolger, 2002). Mediation was considered statistically significant when the 95% confidence interval of the bootstrapped estimate did not include zero (Preacher and Hayes, 2008).

## 4 Results

### 4.1 Sample characteristics

The demographic characteristics of the 1,869 university physical education teachers are presented in Table 1. The sample included 944 male teachers (50.51%) and 925 female teachers (49.49%), indicating a balanced gender distribution. In terms of educational attainment, 391 respondents (20.92%) held a bachelor’s degree or below, 1,239 (66.29%) held a master’s degree, and 239 (12.79%) held a doctoral degree. Regarding professional titles, 426 participants (22.79%) held junior or lower titles, 791 (42.32%) held intermediate titles, 465 (24.88%) held associate senior titles, and 187 (10.01%) held senior titles. With respect to institutional type, 1,121 respondents (59.98%) were from comprehensive universities, 426 (22.79%) from normal universities, and 322 (17.23%) from specialized institutions. The sample therefore reflected diversity in educational background, professional rank, and institutional context.

**TABLE 1:** Demographic characteristics of the sample (*N* = 1,869).

Variable	Category	Frequency	Percentage (%)
Gender	Male	944	50.51
	Female	925	49.49
Education level	Bachelor’s degree or below	391	20.92
	Master’s degree	1,239	66.29
	Doctoral degree	239	12.79
Professional title	Junior or below	426	22.79
	Intermediate	791	42.32
	Associate senior	465	24.88
	Senior	187	10.01
Institution type	Comprehensive university	1,121	59.98
	Normal university	426	22.79
	Specialized institution	322	17.23

### 4.2 Descriptive statistics and measurement reliability

Descriptive statistics and measurement reliability indices for all study variables are presented in Table 2. The mean scores ranged from 2.44 to 2.57, with standard deviations between 0.74 and 0.87, indicating moderate levels of digital teaching demands, technology-related stress, occupational stress, and job burnout among university physical education teachers. Cronbach’s α coefficients demonstrated satisfactory internal consistency, with Digital Teaching Demands showing an α of 0.878, Technology-Related Stress ranging from 0.861 to 0.930 across subdimensions, Occupational Stress ranging from 0.807 to 0.900, and Job Burnout ranging from 0.868 to 0.889.

**TABLE 2:** Descriptive statistics and measurement reliability of the study variables (*N* = 1,869).

Variable	Mean	SD	α	Factor loading	CR	AVE
Digital teaching demands	2.44	0.87	0.878	0.726–0.757	0.880	0.551
Technology-related stress	2.51	0.76	0.861–0.930	0.772–0.775	0.817	0.599
Occupational stress	2.57	0.74	0.807–0.900	0.733–0.785	0.874	0.581
Job burnout	2.51	0.77	0.868–0.889	0.738–0.765	0.799	0.569

SD, standard deviation; α, Cronbach’s alpha; CR, composite reliability; AVE, average variance extracted.

Confirmatory factor analysis results supported acceptable measurement properties. Standardized factor loadings ranged from 0.726 to 0.757 for Digital Teaching Demands, from 0.772 to 0.775 for Technology-Related Stress, from 0.733 to 0.785 for Occupational Stress, and from 0.738 to 0.765 for Job Burnout, exceeding recommended thresholds. Composite reliability (CR) values ranged from 0.799 to 0.880, all above 0.70, and average variance extracted (AVE) values ranged from 0.551 to 0.599, exceeding the 0.50 criterion. These results indicate adequate internal consistency reliability and convergent validity for all constructs.

### 4.3 Common method bias test

Given that all variables were measured using self-report questionnaires, common method bias (CMB) was addressed through both procedural and statistical approaches. During questionnaire administration, several procedural control strategies were adopted to reduce the likelihood of common method variance. Participation was voluntary and anonymous, and respondents were informed that there were no right or wrong answers. The questionnaire instructions emphasized confidentiality and encouraged participants to answer honestly based on their actual teaching experiences. In addition, the items measuring different constructs were presented in separate sections with clear instructions, which helped reduce respondents’ tendency to infer the hypothesized relationships among variables.

Statistical tests were then conducted to further evaluate the potential influence of common method bias. Harman’s single-factor test was first conducted using unrotated exploratory factor analysis. A total of 12 factors with eigenvalues > 1 were extracted, accounting for 65.96% of the cumulative variance. The first factor explained 28.94% of the total variance, which was below the commonly used threshold of 40%. This result suggests that no single factor accounted for the majority of covariance among the measures.

Confirmatory factor analysis was subsequently performed to compare alternative measurement models, including one-factor, two-factor, three-factor, four-factor theoretical model, and an unmeasured latent method construct (ULMC) model. As shown in Table 3, the one-factor model demonstrated poor fit, while the two-factor and three-factor models also failed to reach acceptable fit levels. In contrast, the hypothesized four-factor model showed excellent fit to the data, χ^2^/df = 1.04, CFI = 1.000, TLI = 1.000, SRMR = 0.013, RMSEA = 0.005. The ULMC model showed almost identical fit to the four-factor model and did not produce a meaningful improvement in model fit, indicating that adding a common method factor did not substantially change the measurement structure.

**TABLE 3:** Comparison of competing measurement models and common method bias tests (*N* = 1,869).

Model	χ^2^	df	χ^2^/df	CFI	TLI	SRMR	RMSEA (90% CI)
One-factor	4327.73	119	36.37	0.674	0.627	0.115	0.138 (0.134, 0.141)
Two-factor	3837.65	118	32.52	0.712	0.668	0.110	0.130 (0.126, 0.133)
Three-factor	2041.03	116	17.60	0.851	0.825	0.116	0.094 (0.091, 0.098)
Four-factor	117.83	113	1.04	1.000	1.000	0.013	0.005 (0.000, 0.013)
ULMC-factor	117.83	112	1.05	1.000	0.999	0.013	0.005 (0.000, 0.013)

ULMC, unmeasured latent method construct; CFI, comparative fit index; TLI, Tucker–Lewis index; SRMR, standardized root mean square residual; RMSEA, root mean square error of approximation; CI, confidence interval.

Because the four-factor model produced unusually high incremental fit indices, the AMOS output and model specification were carefully rechecked. The CFA model was estimated using the prespecified indicator structure described in the data analysis section: Digital Teaching Demands was represented by its six original items, whereas Technology-Related Stress, Occupational Stress, and Job Burnout were represented by their theoretically defined subdimension mean scores. Thus, the model included 17 observed indicators in total. No *post-hoc* correlated residuals, cross-loadings, or modification-index-based adjustments were added to force model fit. The very high CFI and TLI values should therefore be interpreted in light of the relatively parsimonious indicator structure, the clear theoretical separation among the four constructs, and the substantially poorer fit of the competing one-, two-, and three-factor models. Overall, the procedural controls and statistical evidence suggest that common method bias did not appear to dominate the observed associations, although the possibility of self-report bias cannot be completely ruled out.

### 4.4 Correlation analysis and discriminant validity

Pearson correlation analyses were conducted to examine the bivariate associations among the study variables. As shown in Table 4, Digital Teaching Demands, Technology-Related Stress, Occupational Stress, and Job Burnout were all positively and significantly correlated in the expected directions. These results provide preliminary support for the hypothesized associations among the four constructs. Discriminant validity was examined using the Fornell–Larcker criterion. The square root of the average variance extracted (AVE) for each construct was greater than its corresponding correlations with other constructs, indicating that each construct shared more variance with its own indicators than with other latent constructs. This pattern supports acceptable discriminant validity.

**TABLE 4:** Correlations and discriminant validity among key variables (*N* = 1,869).

Variable	Digital teaching demands	Technology-related stress	Occupational stress	Job burnout
Digital teaching demands	0.742	0.774	0.762	0.754
Technology-related stress	0.301^***^			
Occupational stress	0.396^***^	0.604^***^		
Job burnout	0.346^***^	0.495^***^	0.538^***^	

^***^p<0.001. Values on the diagonal represent the square roots of the average variance extracted (AVE).

### 4.5 Multiple regression analysis

Hierarchical multiple regression analyses were conducted to further examine the associations among the study variables. As shown in Table 5, the control-variable model was statistically significant. Gender and professional title were significantly associated with job burnout, whereas education level and institution type were not significant predictors. After Digital Teaching Demands, Technology-Related Stress, and Occupational Stress were added to the model, the explained variance in job burnout increased substantially. All three focal predictors were positively and significantly associated with job burnout, providing preliminary evidence consistent with the proposed direct and stress-related pathways. These regression results were used as supplementary evidence before testing the full mediation framework through structural equation modeling.

**TABLE 5:** Hierarchical multiple regression analysis predicting job burnout (*N* = 1,869).

Variables	Model 1		Model 2	
	β	SE	β	SE
Control variables				
Gender	−0.122^***^	0.034	−0.079^***^	0.028
Education level	0.021	0.021	0.003	0.017
Professional title	−0.218^***^	0.015	−0.093^***^	0.013
Institution type	0.023	0.021	0.011	0.017
Independent variable				
Digital teaching demands			0.133^***^	0.018
Mediator				
Technology-related stress			0.249^***^	0.023
Occupational stress			0.307^***^	0.025
Model summary				
*R* ^2^	0.067		0.366	
△ *R*^2^	0.067		0.299	
F	33.213^***^		153.779^***^	

Standardized coefficients (β) are reported.

^***^p < 0.001.

### 4.6 Structural equation modeling analysis

**FIGURE 2:**
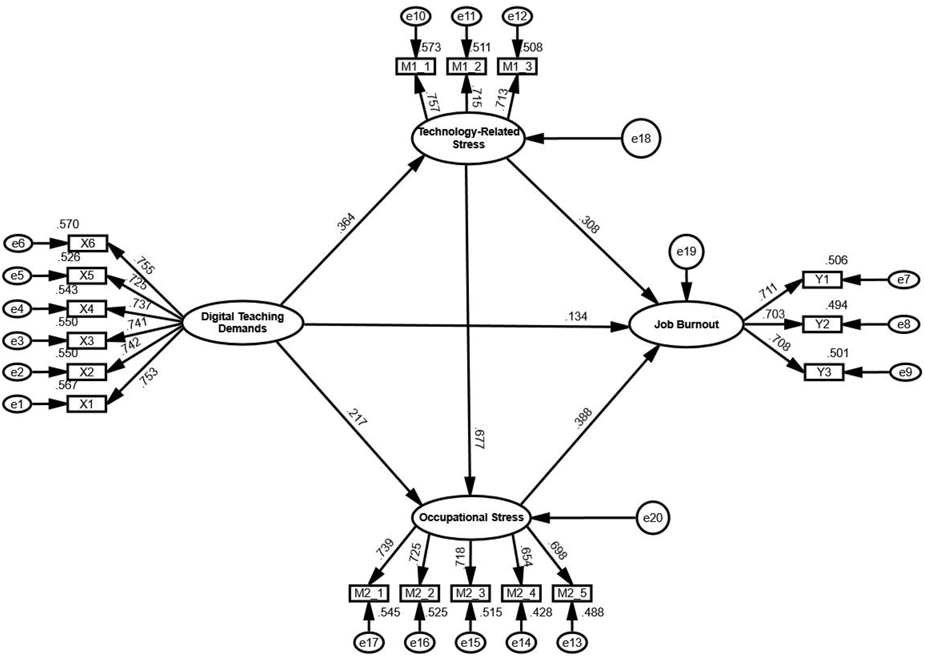
Structural equation model of the relationships among digital teaching demands, technology-related stress, occupational stress, and job burnout.

Structural equation modeling (SEM) was conducted to examine the hypothesized relationships among Digital Teaching Demands, Technology-Related Stress, Occupational Stress, and Job Burnout. The structural model demonstrated satisfactory fit to the data, consistent with the measurement model results. The standardized path coefficients are illustrated in Figure 2.

Digital Teaching Demands was positively associated with Job Burnout (β = 0.134). Digital Teaching Demands was also positively associated with Technology-Related Stress (β = 0.364) and Occupational Stress (β = 0.217). Technology-Related Stress showed a positive association with Job Burnout (β = 0.308), while Occupational Stress was positively associated with Job Burnout (β = 0.388). In addition, Technology-Related Stress was positively associated with Occupational Stress (β = 0.677). These findings indicate that Digital Teaching Demands was related to Job Burnout both directly and through its associations with Technology-Related Stress and Occupational Stress. The structural paths were statistically significant and aligned with the theoretical framework proposed in this study.

### 4.7 Bootstrapping mediation test

The mediation effects were examined using the bias-corrected percentile bootstrap method with 5,000 resamples. The results are presented in Table 6. The total effect of Digital Teaching Demands on Job Burnout was significant (β = 0.426, *p* < 0.001), with a 95% confidence interval that did not include zero, indicating an overall positive association.

**TABLE 6:** Bootstrapping results for direct and indirect effects (*N* = 1,869).

Path	β	Boot SE	Boot LLCI	Boot ULCI	Ratio (%)
Direct effect	0.134^***^	0.029	0.079	0.191	31.46
Indirect effects	0.292^***^	0.019	0.254	0.330	68.54
Digital teaching demands→Technology-related stress→Job burnout	0.112^***^	0.020	0.076	0.154	26.29
Digital teaching demands→Occupational stress→Job burnout	0.084^***^	0.015	0.057	0.116	19.72
Digital teaching demands→Technology-related stress→Occupational stress→Job burnout	0.095^***^	0.014	0.069	0.125	22.30
Total effect	0.426^***^	0.025	0.377	0.474	100

^***^p < 0.001. Boot LLCI, the lower bound of the 95% confidence interval. Boot ULCI, the upper limit of the 95% confidence interval (bias-corrected percentile bootstrap method). Bootstrap sample size = 5,000.

The direct effect remained significant after including the mediators (β = 0.134, *p* < 0.001), accounting for 31.46% of the total effect. The total indirect effect was also significant (β = 0.292, *p* < 0.001), with a 95% confidence interval of [0.254, 0.330], accounting for 68.54% of the total effect.

Regarding specific indirect pathways, the indirect effect through Technology-Related Stress was significant [β = 0.112, 95% CI (0.076, 0.154)], accounting for 26.29% of the total effect. The indirect effect through Occupational Stress was also significant [β = 0.084, 95% CI (0.057, 0.116)], accounting for 19.72% of the total effect. In addition, the indirect pathway through Technology-Related Stress and Occupational Stress was significant [β = 0.095, 95% CI (0.069, 0.125)], accounting for 22.30% of the total effect. None of the confidence intervals included zero, indicating statistically significant mediation effects.

In terms of hypothesis testing, H1 was supported, as Digital Teaching Demands was positively associated with Job Burnout. H2 was supported, given the significant mediating role of Technology-Related Stress. H3 was supported, as Occupational Stress significantly mediated the association. H4 was also supported, as the indirect pathway involving both Technology-Related Stress and Occupational Stress was statistically significant.

## 5 Discussion

### 5.1 Summary of main findings

This study examined the association between digital teaching demands and job burnout among university physical education teachers, with technology-related stress and occupational stress specified as stress-related pathways. Overall, the findings supported the proposed framework. Digital teaching demands were positively associated with job burnout, and this association was reflected largely through technology-related stress and occupational stress. The results also supported a sequential pathway in which digital teaching demands were associated with technology-related stress, which was in turn associated with broader occupational stress and, subsequently, job burnout.

These findings suggest that burnout in digitally intensified PE teaching contexts cannot be fully understood by focusing on digital requirements alone. Rather, digital teaching demands appear to be more relevant to burnout when they are accompanied by technology-specific strain and broader perceptions of work pressure. This pattern is consistent with the view that the wellbeing implications of educational digitalization depend not merely on the presence of digital tools, but on how digital requirements are embedded in teachers’ workload, professional expectations, and institutional support systems.

### 5.2 Digital teaching demands and job burnout

The positive association between digital teaching demands and job burnout is consistent with the health-impairment process proposed by the job demands–resources model. From this perspective, job demands are not problematic simply because they exist; they become consequential when they require sustained effort and are not matched by adequate resources, autonomy, recovery opportunities, or organizational support (Bakker and Demerouti, 2007; Bakker and Demerouti, 2017; Demerouti et al., 2001). The present findings therefore should not be interpreted as evidence that digitalization is inherently harmful. Instead, they suggest that digital teaching requirements may become a risk factor when they accumulate as additional work rather than being integrated into a balanced and supportive work system.

This interpretation is particularly relevant for university PE teachers. PE teaching is characterized by embodied demonstration, real-time movement observation, safety management, venue coordination, and immediate feedback. Digital teaching demands add another layer of work, including online communication, platform-based documentation, digital resource production, data reporting, and adaptation to changing systems. When these requirements are superimposed on physical-space instruction, they may blur work boundaries and intensify role demands. In this sense, the issue is not technology itself, but a potential demands–resources mismatch in which digital expectations expand faster than the time, technical support, training, and institutional coordination needed to manage them.

The findings also highlight the need to treat digital teaching demands as a work-design issue rather than merely as a matter of instructional innovation. Digital tools may improve teaching, assessment, and communication when they are well aligned with pedagogical needs. However, when digital tools multiply reporting channels, extend availability expectations, or require repeated documentation, they may increase perceived strain. For PE teachers, this tension may be especially visible because the core of their work remains physically situated, interactive, and safety-sensitive.

### 5.3 Technology-related stress as a mediating pathway

The findings support technology-related stress as an important pathway linking digital teaching demands with burnout. This result is consistent with technostress research showing that technology overload, technology complexity, technology invasion, uncertainty, and insecurity can undermine wellbeing when digital systems are experienced as intrusive or difficult to manage (Brod, 1984; Pflügner et al., 2024; Ragu-Nathan et al., 2008; Tarafdar et al., 2007). The present study extends this logic to university PE teaching by showing that ICT-related teaching demands are associated with burnout partly through teachers’ appraisals of technology-related strain.

Recent teacher-focused technostress research provides useful context for this finding. Wang and Li (2019) conceptualized technostress among university teachers as a multidimensional person–environment misfit, suggesting that strain arises when teachers perceive a mismatch between technological requirements, personal capabilities, institutional expectations, and the teaching environment. Solís et al. (2023) further showed that perceived organizational support is closely related to teachers’ technostress, indicating that technology-related strain is shaped by organizational conditions as well as individual competence. Levante et al. (2025) linked technostress with emotional exhaustion among teaching staff, while Yang D. et al. (2025) emphasized in a systematic review that teacher technostress research needs to consider both sources of strain and mitigation strategies. These studies align with the present finding that digital demands become more consequential when they are appraised as technology-related pressure.

For university PE teachers, technostress may have a discipline-specific form. Digital requirements may require teachers to translate movement-based instruction into videos, manage online teaching spaces, process activity or fitness data, and communicate with students across multiple platforms. These tasks may demand technical skills that differ from teachers’ embodied pedagogical expertise. In addition, PE classes often occur in gymnasiums, outdoor venues, or other practical teaching spaces where devices, networks, or platforms may be less stable than in conventional classrooms. Such conditions may increase the likelihood that digital teaching requirements are experienced as interruptions, uncertainty, or additional workload rather than as straightforward instructional support.

The technology-related stress pathway also clarifies the distinction between digital requirements and digital strain. Digital teaching demands describe what teachers are expected to do; technology-related stress describes how teachers experience and appraise those expectations. This distinction is important because it avoids treating digitalization as uniformly beneficial or harmful. The same digital requirement may be experienced as manageable when teachers have sufficient time, competence, and support, but as stressful when systems are fragmented, expectations are unclear, or support is insufficient.

### 5.4 Occupational stress as a mediating pathway

Occupational stress also functioned as an important pathway in the association between digital teaching demands and job burnout. This finding suggests that digital teaching demands are not limited to technology-specific frustration; they may also contribute to a broader sense of work pressure. Occupational stress captures the overall strain of teaching work, including workload, role pressure, evaluation demands, professional development pressure, and student-related demands. Thus, while technostress reflects teachers’ strain in relation to digital technologies, occupational stress reflects how digital requirements may become embedded in the wider job context.

This pathway is consistent with the JD–R perspective and with broader work-stress theories. Digital requirements can increase occupational stress when they expand responsibilities, intensify monitoring, or compress time for other professional tasks. Digital platforms may make teaching more visible and traceable, which can support quality assurance, but they may also increase documentation and accountability pressures when institutional expectations are excessive or poorly coordinated. Digital tasks may also extend communication beyond formal working hours, making recovery more difficult. These processes are compatible with the view that burnout develops when sustained job demands exceed available resources and recovery opportunities (Maslach et al., 2001; Sonnentag and Fritz, 2015).

In PE teaching settings, occupational stress may be intensified by the combination of physical instruction and digital administration. PE teachers are responsible for safety, class organization, movement correction, venue management, and student participation. When digital requirements are added to these responsibilities, teachers may experience role expansion rather than task substitution. They are expected not only to teach and supervise physical activities, but also to document teaching processes, upload evidence, manage online communication, and handle digital assessment records. This may help explain why broader occupational stress is a central part of the burnout-related pathway.

The findings also suggest that interventions focused only on technology skills may be insufficient. Training can help teachers use platforms more effectively, but it cannot fully reduce burnout risk if the broader workload structure remains unchanged. Work design factors—such as evaluation criteria, administrative reporting, scheduling, documentation intensity, and recognition of digital labor—are likely to remain important. Therefore, occupational stress should be addressed not only through individual coping strategies but also through institutional workload management and evaluation reform.

### 5.5 Joint mechanism involving technology-related stress and occupational stress

Beyond the two parallel pathways, the findings support a sequential pathway involving both technology-related stress and occupational stress. This pattern suggests that technology-specific strain may spill over into broader work pressure. Technostress may begin with difficulty using platforms, uncertainty about system changes, communication overload, or troubleshooting. Over time, however, these experiences can delay teaching preparation, fragment attention, extend working time, and reduce recovery opportunities. As a result, technology-related strain may become part of a wider occupational stress process.

This sequential logic is consistent with conservation of resources theory, which proposes that resource loss can accumulate and that strain in one domain may reduce an individual’s capacity to cope with demands in another domain (Hobfoll, 1989; Hobfoll et al., 2018). In digitally intensified teaching, time and energy spent managing technological difficulties may reduce the resources available for lesson preparation, student interaction, research responsibilities, and personal recovery. When technology-related stress consumes these resources, teachers may become more vulnerable to broader occupational pressure and eventually to burnout-related symptoms.

The sequential pathway is especially meaningful for university PE teachers because digital work and embodied teaching work often coexist rather than replace each other. PE teachers may complete physically demanding classes during the day and then continue with online communication, video preparation, teaching documentation, or data reporting after class. In this situation, technostress is not isolated from the rest of the job. It can become a pathway through which digital requirements reshape the entire experience of work pressure. This interpretation is also consistent with recent discussions of digital work stress, which emphasize that digital stressors are often embedded in organizational systems rather than confined to individual technology use (Gimpel et al., 2025; Girardi et al., 2025; Yang H.-J. et al., 2025).

At the same time, this sequential interpretation should be treated cautiously because the study used cross-sectional data. The findings are consistent with the proposed theoretical ordering, but they do not establish temporal causality. Future longitudinal or experience-sampling research would be useful for examining whether technology-related stress precedes increases in occupational stress over time and whether changes in these stress processes predict later burnout.

### 5.6 Theoretical implications

Clarifying the pathways linking digital teaching demands to burnout contributes to understanding how digital transformation reshapes job demands and stress processes in a teaching domain characterized by embodied instruction and safety management. The present study offers three theoretical implications.

First, the study conceptualizes ICT-related digital teaching demands as a form of job demand in higher education PE teaching. By focusing on communication overload, accessibility expectations, interruptions, and equipment-related work disruption, the study foregrounds structural aspects of digitally mediated work rather than teachers’ general attitudes toward technology. This contributes to JD–R-based research by showing how digitalization can be examined as part of the health-impairment process. At the same time, the findings imply that this process should be interpreted through a demands–resources lens: digital demands may become problematic when they are not matched by resources such as time, autonomy, technical support, and digital competence.

Second, the study distinguishes technology-specific strain from broader occupational pressure. Technology-related stress captures strain arising from technology use and technology governance, whereas occupational stress captures more general work-pressure appraisals. Modeling these constructs together reduces the risk of attributing burnout simply to “technology.” Instead, the findings suggest that digital teaching demands are associated with burnout through both technology-specific and general occupational stress processes. This distinction helps integrate technostress research with broader occupational stress and burnout theories.

Third, the study extends digitalization and wellbeing research to university PE teachers, a group whose work is shaped by embodied demonstration, physical activity supervision, safety responsibility, and activity-based assessment. The findings suggest that digital transformation in PE should be understood as work redesign, not merely as the adoption of new instructional tools. Digital systems reshape communication, documentation, evaluation, and time boundaries. Therefore, theoretical models of educational digitalization should consider how digital technologies interact with the disciplinary characteristics of teaching work.

The present model focuses on stress-related pathways, but it also points toward a more complete JD–R framework for future research. Digital competence, perceived organizational support, technical support, autonomy, leadership support, and peer collaboration may operate as resources that buffer the association between digital demands and stress. Future studies could examine whether these resources moderate the pathways from digital teaching demands to technostress, from technostress to occupational stress, and from occupational stress to burnout.

### 5.7 Practical implications

The findings suggest that burnout-related risks in digitally intensified teaching contexts are more likely to be reduced through combined interventions targeting digital governance, work design, and teacher support. Practical responses should therefore move beyond asking individual teachers to adapt to technology and should also address the organizational arrangements that make digital work stressful.

At the institutional level, universities should reduce unnecessary complexity in digital teaching systems. Platform integration and process simplification are especially important for PE departments. For example, universities could adopt integrated applications that synchronize wearable-device data, attendance records, physical-fitness test results, and LMS gradebooks, thereby reducing repeated manual entry. Digital systems could also automatically generate student feedback reports from physical-fitness data, provide standardized templates for uploading instructional videos, and archive teaching evidence in a single system rather than requiring teachers to upload the same materials to multiple platforms. Such measures would make digital work more supportive and less disruptive.

At the policy and management level, workload allocation should explicitly recognize the time costs of digital teaching. Producing instructional videos, maintaining online course spaces, responding to platform-based messages, processing activity data, and preparing digital evidence for evaluation all require labor. If these tasks are added without reducing other responsibilities, digitalization may intensify occupational stress. Universities should therefore clarify which digital records are genuinely necessary for teaching quality assurance and avoid excessive documentation that does not contribute meaningfully to student learning or teacher development.

At the evaluation level, digital indicators should be used carefully. Platform data and uploaded teaching artifacts may provide useful evidence of teaching activity, but overreliance on digital traces may undervalue the embodied, interactive, and safety-related dimensions of PE teaching. Evaluation systems should therefore balance digital evidence with discipline-specific teaching quality indicators, such as movement demonstration, risk management, student engagement, individualized feedback, and physical activity participation. This balanced approach may reduce the perception that PE teachers must convert all valuable teaching work into digital artifacts.

At the teacher-support level, training remains necessary but should be task-oriented and context-sensitive. PE teachers may benefit from targeted support on short instructional-video production, LMS assessment functions, wearable-data interpretation, online feedback strategies, and troubleshooting in venue-based or outdoor teaching conditions. Peer mentoring and communities of practice may also reduce perceived complexity and normalize the learning process. However, training alone is unlikely to be sufficient if institutional requirements, workload rules, and platform systems remain fragmented. Sustainable intervention requires aligning digital expectations with organizational resources.

### 5.8 Limitations and directions for future research

Several limitations should be considered when interpreting the findings. First, the study used cross-sectional survey data. Although the proposed model is theoretically grounded and the results are consistent with the hypothesized pathways, the findings should be interpreted as associations rather than causal effects. Longitudinal designs, experience-sampling methods, or intervention studies would be better suited for examining temporal ordering and changes in stress processes during digital transformation.

Second, the sample was drawn from universities in Henan Province, China. This regional focus provides a meaningful context for examining university PE teachers, but it may limit the generalizability of the findings. Digital infrastructure, institutional funding, platform integration, technical support, workload policies, and evaluation systems may differ across regions and countries. In more digitally developed regions, teachers may face stronger platform-based evaluation and data-reporting demands, but they may also receive better technical support. In less digitally developed regions, digital requirements may be less intensive, but unstable systems or limited support could amplify technostress. Future research should compare different regional and institutional contexts to examine whether the strength of these pathways varies across digital governance environments.

Third, the study operationalized digital teaching demands using the ICT Demands at Work scale. This scale captures important ICT-related work demands, including communication overload, accessibility expectations, interruptions, and equipment malfunction. However, it does not fully capture PE-specific digital teaching demands, such as producing movement-demonstration videos, managing wearable-device data, using app-based activity monitoring, or integrating real-time performance data into teaching assessment. Future studies should develop and validate a PE-specific Digital Teaching Demand Scale that reflects the embodied, data-based, and venue-dependent characteristics of PE teaching.

Fourth, all measures were self-reported. Although procedural controls, Harman’s single-factor test, competing CFA models, and the ULMC comparison suggested that common method bias did not appear to dominate the observed associations, self-reporting may still be influenced by social desirability, response style, or subjective perceptions of stress and burnout. Future studies could combine self-report data with objective or multi-source indicators, such as platform-use records, workload logs, teaching schedules, peer evaluations, or qualitative interviews.

Fifth, the present model focused on demands and stress pathways and did not explicitly include protective resources. Future research could extend the model by incorporating digital competence, perceived organizational support, technical support, autonomy, leadership support, and peer collaboration as moderators. A moderated mediation model would allow researchers to examine when digital teaching demands are most likely to translate into technostress, occupational stress, and burnout, and under what resource conditions these associations may be weakened. In addition, although the present study adapted relevant teacher stress and burnout instruments to the university PE context, future research could further validate measurement tools specifically designed for university PE teachers.

## 6 Conclusion

Based on a sample of 1,869 university physical education teachers from Henan Province, China, this study found that ICT-related digital teaching demands were positively associated with job burnout. This association was reflected largely through technology-related stress and occupational stress, indicating that both technology-specific strain and broader occupational pressure are relevant to understanding burnout in digitally intensified PE teaching contexts. The findings support the proposed stress-related pathway model and suggest that university digital teaching initiatives should address not only teachers’ technology use, but also the work-design conditions under which digital requirements are implemented. Given the cross-sectional design, these findings should be interpreted as associations, and future longitudinal research is needed to further examine their temporal ordering.

## Data Availability

The original contributions presented in this study are included in the article/Supplementary material, further inquiries can be directed to the corresponding author.
